# Deep Reinforcement Learning with Local Attention for Single Agile Optical Satellite Scheduling Problem

**DOI:** 10.3390/s24196396

**Published:** 2024-10-02

**Authors:** Zheng Liu, Wei Xiong, Chi Han, Xiaolan Yu

**Affiliations:** National Key Laboratory of Space Target Awareness, Space Engineering University, Beijing 101416, China; wei_xiong@hgd.edu.cn (W.X.); hanchi@hgd.edu.cn (C.H.); yuxiaolan@hgd.edu.cn (X.Y.)

**Keywords:** single agile optical satellite scheduling, deep reinforcement learning, local attention, adaptive learning rate

## Abstract

This paper investigates the single agile optical satellite scheduling problem, which has received increasing attention due to the rapid growth in earth observation requirements. Owing to the complicated constraints and considerable solution space of this problem, the conventional exact methods and heuristic methods, which are sensitive to the problem scale, demand high computational expenses. Thus, an efficient approach is demanded to solve this problem, and this paper proposes a deep reinforcement learning algorithm with a local attention mechanism. A mathematical model is first established to describe this problem, which considers a series of complex constraints and takes the profit ratio of completed tasks as the optimization objective. Then, a neural network framework with an encoder–decoder structure is adopted to generate high-quality solutions, and a local attention mechanism is designed to improve the generation of solutions. In addition, an adaptive learning rate strategy is proposed to guide the actor–critic training algorithm to dynamically adjust the learning rate in the training process to enhance the training effectiveness of the proposed network. Finally, extensive experiments verify that the proposed algorithm outperforms the comparison algorithms in terms of solution quality, generalization performance, and computation efficiency.

## 1. Introduction

Agile optical satellites (AOSs), as a new generation of optical satellites, have superior attitude maneuverability, which can adjust attitude flexibly and quickly on the three axes of pitch, roll and yaw. Over past years, they have played increasingly significant roles in many fields, such as environmental monitoring, marine development, agricultural production, city planning, and military reconnaissance [1,2]. The agile characteristics improve the observation capability of AOSs but also lead to a more complex attitude maneuver process. With the surge in earth observation demand, scheduling an agile optical satellite has become a challenging problem.

**Figure 1:**
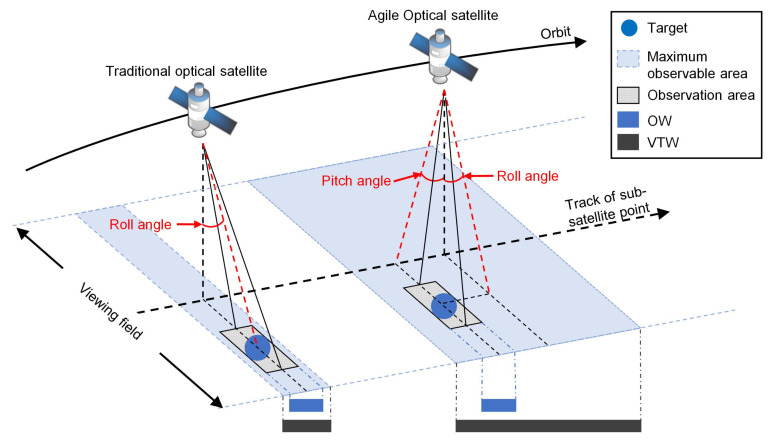
Observation process of the traditional optical satellite and AOS.

Compared with traditional optical satellites, AOSs can observe the target during a much longer observable period due to its ability to rotate around the pitch axis, as shown in Figure 1. The period during which the satellite can observe the target is called the visible time window (VTW), and the duration of the actual observation event is called the observation window (OW). In this study, the AOS adopts a common push-broom observation mode [3] and adjusts its attitude only by rolling and pitching. In the observation process of an AOS, its pitch angle determines the start time of the OW, and its roll angle is determined by the relative position of the AOS and the target. Thus, in a VTW, the pitch angle is variable but the roll angle can be regarded as a fixed value. When an AOS observes two adjacent targets, a short period is needed for the attitude adjustment, which is called the transition time. The time interval between two successive observation actions must not be less than their required transition time [4]. Only if the observation time of the previous task is determined can the observation time of the next one be determined. Therefore, the observation time of multiple tasks must be determined one by one, which is a sequential decision process.

The purpose of the single AOS scheduling problem (SAOSSP) is to reasonably arrange the observation sequence and observation time of tasks [3]. The SAOSSP has been proven as an NP-hard problem [5], exhibiting the characteristics of a combination explosion, and its solving time grows exponentially with the problem scale [6]. The complexity of this problem is mainly reflected in three aspects. First, each target may have several VTWs in different orbits, causing multiple possibilities of the VTW selection, and the observation event can start at any time within the selected VTW, causing various optional possibilities of the OW. Second, owing to the complicated constraints and the considerable solution space, an enormous number of constraint-checking steps and a thorough search in the solution space are needed [7], demanding high computational expenses. Third, this problem is typically an oversubscribed scheduling problem where only partial tasks have the chance to be accomplished [8,9], and the increase of the problem scale can further expand its computational complexity. Therefore, the SAOSSP is hard and complex to solve, and an efficient scheduling method is badly required to accomplish the more observation tasks and reach the maximum observation profit.

Over the past few decades, extensive research has been carried out, and many contributions have been made to solve the agile satellite scheduling problem. Existing approaches can be roughly distributed into three categories: exact algorithms, heuristic algorithms and deep reinforcement learning (DRL) algorithms. Some researchers adopt exact algorithms to solve this problem. Lemaître et al. [5] gave the general description of the agile satellite scheduling problem for the first time and proposed a dynamic programming algorithm. Chu et al. [10] presented a branch and bound algorithm with a look-ahead method and three pruning strategies to tackle the simplified problem in a small-size instance. Peng et al. [11] considered the time-dependent profits and presented an adaptive-directional dynamic programming algorithm with decremental state space relaxation for the single-orbit scheduling of the single agile satellite. The above studies summarily prove that exact algorithms can explore the whole search space and obtain the optimal solution, and they are applicable to the single-orbit or small-scale satellite scheduling. However, as the problem scale expands, the computational cost of the exact algorithms tends to be unacceptable because of the NP-hard characteristic and the complex constraints.

Differing from exact algorithms, heuristic algorithms can iteratively search for a good solution with relatively lower computational cost, and they have been extensively applied to the agile satellite scheduling due to the excellent abilities of exploration and exploitation, such as genetic algorithms (GAs) [12,13], particle swarm optimization (PSO) algorithms [14,15], ant colony optimization (ACO) algorithms [16,17], artificial bee colony (ABC) algorithms [18] and so on. Chatterjee and Tharmarasa [19] formulated a mixed-integer non-linear optimization model and proposed an elitist mixed coded genetic algorithm algorithm to solve the agile satellite scheduling problem. Du et al. [20] considered the drift angle constraint of the observation instrument and developed an improved ant colony algorithm based on a sensational and consciousness strategy to solve the area target observation problem of agile satellites. Yang et al. [21] improved the three search phases of the basic ABC algorithm and presented a hybrid discrete artificial bee colony (HDABC) algorithm to address the satellite scheduling problem. These heuristic algorithms can optimize solutions through superior search mechanisms and iterative updates of population, but the issue of considerable computational time and the convergence problem still exist.

To avoid the above shortcomings, researchers demand for a novel, efficient, and non-iterative method to solve the agile satellite scheduling problem. With the development of artificial intelligence technology, deep reinforcement learning (DRL) methods have achieved excellent results in many fields [22,23,24,25], especially in large-scale combination optimization problems [26,27]. Because the agile satellite scheduling problem can be formulated as a sequential decision problem [28,29], DRL algorithms can be adopted to replace heuristic algorithms, which can directly generate solutions without an iterative search. Chen et al. [30] developed an end-to-end network model with a convolution neural network (CNN) as the encoder, a gated recurrent unit (GRU) as the decoder, and an attention mechanism to obtain the relevance between the output task and each input task, and this model achieved good results in the small-scale scheduling instances. Zhao et al. [31] proposed a two-phase neural combination optimization method, which adopted a Pointer Network (PtrNet) to generate a permutation of executable tasks and a deep deterministic policy gradient algorithm to determine the observation time of selected tasks. Wei et al. [32] proposed a DRL and parameter transfer-based approach (RLPT), consisting of a task encoder composed of a GRU, a feature encoder composed of a CNN, and a decoder composed of an attention mechanism. These studies have preliminarily verified the feasibility and effectiveness of DRL, but there are still some weaknesses. First, the neural networks in these methods can only deal with the inputs of fixed dimensions, and no effective approach is provided to handle the inputs of variable dimensions, especially VTWs, the number of which may be different for different tasks. Second, the above methods based on the encoder–decoder structure all adopt the global attention mechanism whose computation is a bit large, and they do not pay attention to the internal connections among tasks. Third, the above studies only train and validate the proposed methods on the instances of a certain scale, and they do not really apply them to the large-scale problems. Therefore, it is necessary to further explore the improvement of DRL to overcome these deficiencies and efficiently solve the SAOSSP.

In this study, we propose a deep reinforcement learning algorithm with a local attention mechanism (DRLLA) to solve the SAOSP in a non-iterative manner. The primary contributions are summarized as follows:

(1) A neural network framework based on an encoder–decoder structure is designed to construct a high-quality scheduling solution in an auto-regression manner. Furthermore, a local attention mechanism is proposed to guide the network model to focus on the more promising tasks in the solution construction process by narrowing the candidate task range into a local one, which greatly improves the solution quality.
(2) An adaptive learning rate strategy is developed to guide the actor–critic training algorithm to train the proposed network model. In the training process, the adaptive learning rate strategy appropriately reduces or increases the learning rate of the training algorithm according to the reward curve trend, obtaining better training effectiveness.
(3) Extensive experiments are conducted to verify the proposed algorithm with our created SAOSSP datasets, which contain numerous various-scale instances and can be publicly available for related studies. The experimental results validate that the proposed algorithm has superior performance in solution quality, generalization performance, and computational efficiency over the comparison algorithms.

The remainder of this paper is organized as follows. In Section 2, we describe the SAOSSP in detail and build the corresponding mathematical model with complicated constraints and an optimization objective. Section 3 presents a neural network with a local attention mechanism for the SAOSSP and an actor–critic training algorithm with an adaptive learning rate for the training of the proposed network. Section 4 presents the experimental results, and Section 5 presents the conclusions of the study with a summary and directions for future work.

## 2. Problem Description and Model

Based on the previous literature, the SAOSP can be described as a combination optimization problem that aims to arrange the observation sequence of candidate tasks and determine their observation time in the selected VTWs to gain the maximum observation profit. In this section, a detailed description is given and a mathematical model of SAOSP is built with complex constraints and an optimization objective.

### 2.1. Assumptions

In practice, AOS scheduling is a complicated process with a series of practical constraints. In order to simplify and standardize the SAOSSP, it is necessary to make some reasonable assumptions based on the actual engineering background and the previous literature [33,34,35,36,37], which are listed as follows:

(1) The AOS observes only one target every time;
(2) All tasks are point targets or small strip targets which can be observed in one pass;
(3) All tasks are executed at most once;
(4) At the initial moment of each orbital cycle, the AOS completely releases the memory space for the storage of observation data, and its energy for the observation and attitude maneuver is full;
(5) The influence of cloud cover is not considered.

### 2.2. Notations and Variables

The inputs of the SAOSSP contain task requirements and satellite parameters, and the parameters of VTWs are calculated according to the target location and the orbital parameters of the AOS. The output solutions contain the permutation of executable tasks and the corresponding observation action sequence of the AOS. The variables which are used to describe the SAOSSP are defined in Table 1.

In addition to the above variables, several other concepts and variables are involved in this problem. Firstly, the attitude adjustment on each axis can be simplified as “constant acceleration—constant speed—constant deceleration” or “constant acceleration—constant deceleration” [38], and the transition time tranT(·) is formulated as below:

(1) $$tranT\left(\Delta A\right)=\left\{\begin{array}{c} 0,\Delta A=0\\ 2\cdot \sqrt{\frac{\Delta A}{a}}+ST,0<\Delta A⩽\frac{\omega^{2}}{a}\\ \frac{\Delta A}{\omega }+\frac{\omega }{a}+ST,\Delta A>\frac{\omega^{2}}{a} \end{array}\right.,$$

where ΔA is the adjusted angle value on one axis, *a* is the angular acceleration value, *ω* is the maximum angular velocity value, and *ST* is the stability time after adjustment. If the roll angle changes by Δr and the pitch angle changes by Δp, the total transition time ΔT will be ΔT=tranT(Δr)+tranT(Δp).

Secondly, in the VTW $w_{i}^{k}$, the relationship between the pitch angle ${pa}_{i}^{k}$ and the observation start time ${ost}_{i}^{k}$ can be formulated as a linear function [39]:

(2) $${pa}_{i}^{k}=\alpha_{i}^{k}\cdot {ost}_{i}^{k}+\beta_{i}^{k},$$

where $\alpha_{i}^{k}=\frac{-2\cdot p_{max}}{{et}_{i}^{k}-d_{i}{-st}_{i}^{k}}$, and $\beta_{i}^{k}=\frac{{et}_{i}^{k}-d_{i}{+st}_{i}^{k}}{{et}_{i}^{k}-d_{i}{-st}_{i}^{k}}\cdot p_{max}$.

Thirdly, the image quality is determined by the observation start time [4], so we respectively define ${est}_{i}^{k}$ and ${lst}_{i}^{k}$ as the earliest start time and the latest start time meeting the minimum image quality requirement in the VTW $w_{i}^{k}$. In addition, the earliest start time ${est}_{i}^{k}$ corresponds to the maximum pitch angle ${px}_{i}^{k}$ meeting the minimum image quality requirement, and the latest start time ${lst}_{i}^{k}$ corresponds to the minimum one ${px}_{i}^{k}$. All of them can be formulated as below:

(3) $${est}_{i}^{k}={st}_{i}^{k}+\left(q_{i}-1\right)\cdot \frac{{et}_{i}^{k}-d_{i}{-st}_{i}^{k}}{18},$$

(4) $${lst}_{i}^{k}={et}_{i}^{k}-d_{i}-\left(q_{i}-1\right)\cdot \frac{{et}_{i}^{k}-d_{i}{-st}_{i}^{k}}{18},$$

(5) $${px}_{i}^{k}=\alpha_{i}^{k}\cdot {est}_{i}^{k}+\beta_{i}^{k},$$

(6) $${pn}_{i}^{k}=\alpha_{i}^{k}\cdot {lst}_{i}^{k}+\beta_{i}^{k}.$$

Finally, three decision variables are defined to establish the mathematical model of the SAOSSP, which is shown in the following formulae:

(7) $$x_{i}^{k}=\left\{\begin{array}{c} 1,t_{i}\;\mathrm{is}\;\mathrm{successfully}\;\mathrm{executed}\;\mathrm{in}\;w_{i}^{k}\\ 0,\mathrm{otherwise} \end{array}\right.,$$

(8) $$y_{ij}=\left\{\begin{array}{c} 1,t_{i}\;\mathrm{is}\;\mathrm{closely}\;\mathrm{previous}\;\mathrm{to}\;t_{j}\\ 0,\mathrm{otherwise} \end{array}\right.,$$

(9) $$z_{i}^{l}=\left\{\begin{array}{c} 1,t_{i}\;\mathrm{is}\;\mathrm{executed}\;\mathrm{in}\;\mathrm{the}\;\mathrm{orbit}\;o_{l}\\ 0,\mathrm{otherwise} \end{array}\right..$$

### 2.3. Mathematical Formulation

The mathematical formulation of the SAOSSP is defined as follows, where the optimization objective is to maximize the profit ratio of the completed tasks and complicated constraints are taken into consideration.

(10) $$\mathrm{Maximize}\;F=\frac{\sum_{i=1}^{\left|T\right|}\left(p_{i}\cdot \sum_{k=1}^{|W_{i}|}x_{i}^{k}\right)}{\sum_{i=1}^{\left|T\right|}p_{i}},$$

subject to

(11) $$\;\sum_{k=1}^{|W_{i}|}x_{i}^{k}\leq 1,\forall t_{i}\in T;$$

(12) $$\left[{st}_{i}^{k},{et}_{i}^{k}\right]\subseteq OL\left(o_{l}\right),\mathrm{if}\;c_{i}^{k}=l;$$

(13) $${{est}_{i}^{k}\leq ost}_{i}^{k}\leq {lst}_{i}^{k},\mathrm{if}\;x_{i}^{k}=1;$$

(14) $${ost}_{j}^{v}-{oet}_{i}^{u}\geq tranT\left(\left|{pa}_{j}^{v}-{pa}_{i}^{u}\right|\right)+tranT(|{ra}_{j}^{v}-{ra}_{i}^{u}\left|\right),\mathrm{if}\;y_{ij}=1;$$

(15) $$rm\cdot \;\sum_{i=1}^{\left|T\right|}\left(d_{i}\cdot z_{i}^{l}\right)\leq M,\forall o_{l}\in O;$$

(16) $$\;\sum_{j=1}^{\left|T\right|}z_{j}^{l}\left[rea\;\sum_{i=1}^{\left|T\right|}y_{i}^{j}\left(\left|{pa}_{j}^{v}-{pa}_{i}^{u}\right|+\left|{ra}_{j}^{v}-{ra}_{i}^{u}\right|\right)+reo\cdot d_{j}\right]\leq E,\forall o_{l}\in O.$$

Equation (11) denotes the execution uniqueness constraint that every task is executed at most once. Equation (12) denotes the lighting constraint that every candidate VTW must meet the lighting condition. Equation (13) denotes the image quality constraint that the observation start time must satisfy the minimum image quality requirement. Equation (14), where *u* and *v* separately denote the VTW indexes of the two tasks, indicates the transition time constraint that the time interval between two observation actions must be sufficient for attitude adjustment. Equation (15) denotes the memory constraint whereby the total memory consumption for observation cannot exceed the maximum in each orbit cycle. Equation (16) denotes the energy constraint whereby the total energy consumption for attitude adjustment and observation cannot exceed the maximum in each orbit cycle.

## 3. Method

In this section, we propose a deep reinforcement learning algorithm with a local attention mechanism (DRLLA) to address the SAOSSP. The architecture of the designed neural network, its crucial components, and a training approach are described in turn.

### 3.1. Architecture of the Proposed Neural Network

**Figure 2:**
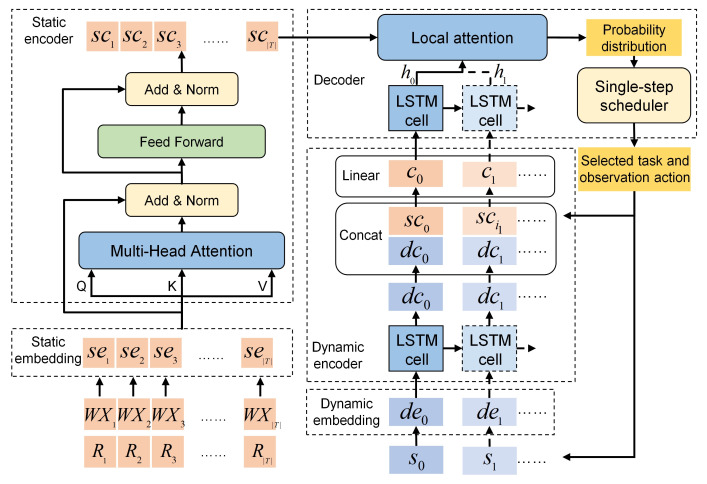
Architecture of the proposed neural network. For task $t_{i}$, ${se}_{i}$ is its static embedding form, and ${sc}_{i}$ is its static encoding form. At step *n*, ${de}_{n}$ is the dynamic embedding form mapped by $s_{n}$, and ${dc}_{n}$ is its dynamic encoding form. $c_{n}$ is the output of the dynamic encoder. $h_{n}$ is the hidden state of the LSTM cell in the decoder.

The architecture of the proposed neural network is depicted in Figure 2, which is an end-to-end framework with an encoder–decoder structure. The neural network is composed of five components: a static embedding layer, a static encoder, a dynamic embedding layer, a dynamic encoder and a decoder. In addition, a local attention mechanism is proposed to improve the generation of solutions.

In the general SAOSSP, given a set of tasks $T=\{t_{i}|i=1,\cdots ,|T|\}$, the neural network is used as the policy network to extract features from inputs and generate a permutation of the executable tasks and a corresponding observation action sequence in an auto-regression way, both of which compose the solution of the problem. The inputs of this problem comprise two parts: task information and satellite state information. Task information is static and consists of time window information and requirement information. For the task $t_{i}$, its time window information is denoted by a set ${WX}_{i}=\left\{{wx}_{i}^{k}=\left[c_{i}^{k},{est}_{i}^{k},{lst}_{i}^{k},{px}_{i}^{k},{pn}_{i}^{k},r_{i}^{k}\right]\left|k=1,\cdots ,\right|W_{i}|\right\}$, and its requirement information is denoted by a vector $R_{i}=\left[d_{i},p_{i}\right]$. Satellite state information is dynamic and changes every time a task is executed. After the AOS executes the task $t_{i}$ through the observation action $a_{i}^{k}$, its state information is denoted as a vector $s_{n}=\left[c_{i}^{k},{oet}_{i}^{k},{pa}_{i}^{k},ra_{i}^{k},{mr}_{i},{er}_{i}\right]$, where *n* is the step number and the observation end time ${oet}_{i}^{k}$ is also the start time of the free state. The output permutation of the executable tasks is denoted as $\left\{i_{n}|n=1,\cdots ,N\right\}$, where $i_{n}$ is the task index and *N* is the length of output permutation. Accordingly, the observation action sequence of the AOS is $A=\{a_{i_{n}}^{k_{i_{n}}}|n=1,\cdots ,N\}$. Obviously, the elements of $s_{n}$ can be obtained in $a_{i_{n}}^{k_{i_{n}}}$. To better utilize the above input information, the designed neural network must be able to process both static and dynamic information simultaneously. Therefore, the proposed neural network adopts a static embedding layer and a static encoder to handle the static inputs and a dynamic embedding layer and a dynamic encoder to deal with the dynamic inputs on the basis of the basic encoder–decoder structure. The above inputs have been normalized before they are formally inputted.

Formally, the set of inputs is denoted as $X=\{{tx}^{i}|i=1,\cdots ,|T|\}$. Each input ${tx}^{i}$ is denoted as a sequence of tuples $\left\{{tx}_{n}^{i}=\left(sx^{i},dx_{n}^{i}\right)|n=0,\cdots ,N\right\}$, where $sx^{i}=\left({WX}_{i},R_{i}\right)$ is the static element, and $dx_{n}^{i}=s_{n}$ is the dynamic one at the decoding step *n*. In addition, $X_{n}$ denotes the state set of all inputs at the decoding step *n*, and $X_{0}$ is the initial state of the inputs. $Y=\{{ty}_{n}|n=0,\cdots ,N\}$ is the final permutation, where ${ty}_{0}$ is an initial virtual tuple composed of the virtual static elements and the initial satellite state vector. $Y_{n}=\left\{{ty}_{0},\cdots ,{ty}_{n}\right\}$ is the decoded permutation up to the step *n*.

At every decoding step n(n=0,⋯,N), the neural network generates the probability distribution of inputs in $X_{n}$ at first. Then, ${ty}_{n}$ points to an available input ${ty}_{n+1}\in X_{n}$ with the highest probability which is chosen as the input of the next decoding step n+1 and added into $Y_{n+1}$, and $X_{n}$ is updated to $X_{n+1}$ according to the constraints of the actual problem, as formulated in the following equations:

(17) $${ty}_{n+1}=\underset{ty}{\arg\max\;}P\left(ty|Y_{n},X_{n};\theta \right),$$

(18) $$X_{n+1}=f\left({ty}_{n+1},X_{n}\right),$$

where f(·) is the state transition function to update the state set of inputs, and *θ* are learnable parameters.

The above process continues until all the available tasks are completed, and the probability chain rule is adopted to factorize the probability of generating the sequence *Y* as

(19) $$P\left(Y|X_{0};\theta \right)=\prod_{n=0}^{N}P\left({ty}_{n+1}|Y_{n},X_{n};\theta \right).$$

The reward of the final scheduling results is the profit ratio of the completed tasks, which is denoted by $R_{a}\left(Y\right)$.

### 3.2. Local Attention Mechanism

The decoding process of the neural network is dynamic, and every time, one task is selected through an attention mechanism according to the static encoding information and the current dynamic encoding information, which utilizes auto-regression. The attention mechanism is adopted to calculate the probability distribution of candidate tasks, and that with the highest probability is selected. In this process, the selection order of tasks represents their execution order. Notably, the unreasonable sorting order can form a low-quality solution.

**Figure 3:**
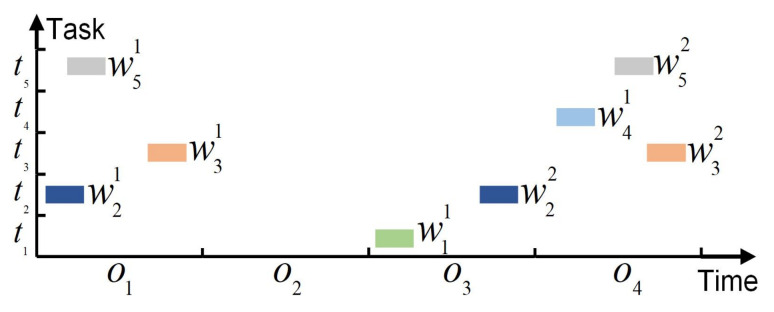
VTWs of five tasks in the four orbits.

**Figure 4:**
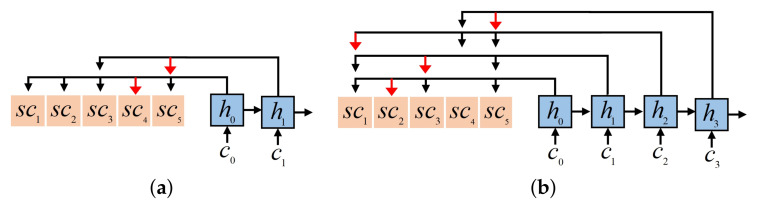
Comparison between global attention and local attention. Under the same circumstances, the global attention mechanism needs more computation. However, it tends to generate unreasonable task orders, leading some tasks to be abandoned, so its decoding step number is less. (**a**) Global attention. At the first step, $t_{4}$ is selected from all the tasks to be executed. $t_{1}$ and $t_{2}$ are deleted since they have no VTWs in the following orbits, and $t_{3}$ and $t_{5}$ are the remaining tasks. At the second step, $t_{5}$ is selected, and $t_{3}$ is deleted. The total step number is 2. (**b**) Local attention. At the first step, $t_{1}$, $t_{2}$, $t_{3}$, and $t_{5}$ have VTWs in the orbits $o_{1}$ and $o_{3}$, and $t_{2}$ is selected from them to be executed. At the second step, $t_{1}$, $t_{3}$, and $t_{5}$ are still available in these two orbits, and $t_{3}$ is selected from them, causing the scheduling in $o_{1}$ to be finished. At the third step, $t_{1}$, $t_{4}$, and $t_{5}$ with VTWs in the next two orbits need to be scheduled, and $t_{1}$ is selected. At the last step, only $t_{4}$ and $t_{5}$ have VTWs in the last orbit $o_{4}$. $t_{5}$ is selected, and $t_{4}$ is deleted. The total step number is 4.

In the previous research [30,31,32], a global attention mechanism is adopted to calculate the probability distribution of all unselected tasks and make neural networks focus on the essential parts of global task information. However, as the scale of the problem expands, the length of the task sequence also becomes longer, making it more difficult for the global attention mechanism to find out the best candidate task quickly and accurately at every decoding step. In the early training process, the neural network has not formed the optimal decision policy, which is highly likely to generate unreasonable sorting orders. Once the task that should have been executed last is selected first, others will lose the opportunity to be executed. For example, Figure 3 shows several VTWs of five tasks in the four orbits. The orbit $o_{2}$ is not considered since no VTW exists in this orbit. As shown in Figure 4a, the global attention mechanism is applied to this example, and all the unselected tasks are included in the candidate range at every decoding step. If $t_{4}$ is selected to be executed in the VTW $w_{4}^{1}$, the tasks without later VTWs will have no chance to be completed, and $t_{1}$ and $t_{2}$ will be abandoned, which could have been executed in the previous orbits. Owing to the tendency to generate unseasonable sorting orders, it is hard for the global attention mechanism to generate great solutions in the training process, making it difficult for the neural network to learn excellent experiences and further influencing the training effect.

To avoid these deficiencies, we propose a local attention mechanism that reduces the range of candidate tasks and focuses on the local task information. An ideal scheduling solution can fully utilize orbit resources and arrange as many feasible tasks as possible in an orbit without causing conflicts. Thus, the range of candidate tasks is limited to the current several orbital cycles at every decoding step, and the local attention mechanism only considers the unselected tasks that satisfy the condition of having VTWs in this local range. The local attention is set to only consider the unselected tasks with VTWs in the current orbit or the next one at every decoding step. As illustrated in Figure 4b, the local attention is employed for the above example. Only $t_{1}$, $t_{2}$, $t_{3}$, and $t_{5}$, which have VTWs in $o_{1}$ or $o_{3}$, are taken into account at the first decoding step. If no unselected tasks have VTWs in the current orbit, those with VTWs in the following two orbits will be considered. If there is only one orbit left, the local attention will only need to focus on the left tasks in this orbit. In this way, the scheduling solutions can be significantly improved. For one thing, it is easier to find the best candidate task within a local range. For another, the observation time of the next task can be arranged close to that of the previous one, preventing some tasks from losing their observation opportunities.

In the proposed local attention mechanism, only the conditional probabilities of partial tasks need to be calculated. Let ${LS}_{n}$ be the set of encoded static elements of tasks meeting the above condition at the decoding step *n*, and $|LS_{n}|$ is the number of these tasks. The conditional probabilities can be calculated as follows:

(20) $$u_{n}=v^{T}\tanh\left(W_{a}\left(h_{t}\right)+W_{b}\left({sc}_{i}\right)\right),sc_{i}\in {LS}_{n};$$

(21) $$P\left(\cdot |Y_{n},X_{n}\right)=\mathrm{softmax}\left(u_{n}\right).$$

In Equations (20) and (21), $v^{T}$, $W_{a}$, and $W_{b}$ are learnable parameters of the local attention layer.

### 3.3. Compositions of the Neural Network

The neural network consists of a static embedding layer, a static encoder, a dynamic embedding layer, a dynamic encoder, and a decoder, which are elaborated in detail below.

#### 3.3.1. Static Embedding Layer

The static embedding layer is used to embed the static elements, including time window information and requirement information, into a high-dimensional vector space. The static elements have the following characteristics: first, the number of tasks is variable; second, the input task sequence does not have an apparent sorting order. However, the features of the time window information and the requirement information are distinct. The VTW number of different tasks is not a fixed value, and the time window information of a task is a time-related sequence. The requirement information of every task is a vector with fixed dimensions. Therefore, the static embedding layer must be able to process task window information and requirement information separately.

**Figure 5:**
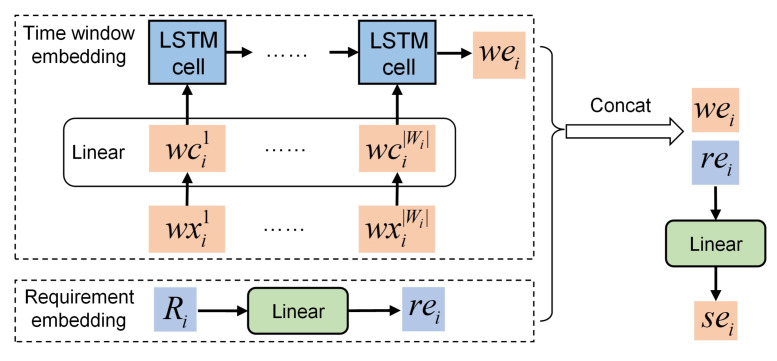
Structure of the static embedding layer.

The structure of the static embedding layer is shown in Figure 5. For $t_{i}$, its time window set ${WX}_{i}$ is embedded through a fully connected network ($W_{w}$) and an LSTM network ($L_{w}$) successively, and a vector ${we}_{i}$ is obtained. Meanwhile, its requirement vector $R_{i}$ is embedded through a fully connected network ($W_{r}$), and a vector ${re}_{i}$ is obtained. Then, the concatenated form of ${we}_{i}$ and ${re}_{i}$ is further processed through a fully connected network ($W_{s}$). The concatenating operator is denoted by Concat(·,·). The final embedding output of *T* is denoted as $SE=\{{se}_{i}|i=1,\cdots ,|T|\}$. The calculating process of ${se}_{i}$ is formulated as follows:

(22) $$we_{i}=L_{w}\left(W_{w}(WX_{i})\right),$$

(23) $$re_{i}=W_{r}\left(R_{i}\right),$$

(24) $$se_{i}=W_{s}\left(\mathrm{Concat}\left(we_{i},re_{i}\right)\right),$$

where $W_{w}$, $L_{w}$, and $W_{s}$ are learnable parameters of the corresponding networks. With the static embedding layer, the proposed neural network can handle the inputs of variable dimensions.

#### 3.3.2. Static Encoder

The static encoder is used to extract features from the static inputs for the subsequent solution construction. Because the input order of tasks is meaningless for this problem, position coding and recurrent neural networks are inapplicable to encoding. Thus, a multi-head self-attention sub-layer ($W_{m}$) and a fully connected feed-forward sub-layer ($W_{f}$) are adopted in the static encoder. The multi-head self-attention mechanism [27] can divide the input sequence into multiple sub-sequences and perform self-attention on each sub-sequence. The self-attention extracts features by calculating the correlation among tasks, and the extracted features of a task contain not only its own feature information but also global feature information. Each sub-sequence corresponds to a head, and different heads focus on different aspects of the input sequence. This mechanism allows the network model to capture different relationships between tasks in the input sequence and efficiently extract features from a long sequence.

To improve the training efficiency and performance, each sub-layer adds a skip-connection structure [40] to alleviate the gradient disappearance problem and a layer-normalization operator [40] to stabilize the training process. The layer-normalization operator is denoted by LN(·), the output of the multi-head self-attention layer is denoted by MH, and the final encoding output of SE is denoted by SC. The static encoding process is formulated as below:

(25) $$MH=\mathrm{LN}\left(SE+W_{m}(SE)\right),$$

(26) $$SC=\mathrm{LN}\left(MH+W_{f}(MH)\right),$$

where $W_{m}$ is the learnable parameter set of the multi-head attention layer, and $W_{f}$ is the learnable parameter set of the fully connected feed-forward layer.

#### 3.3.3. Dynamic Embedding Layer

In the dynamic embedding layer, a fully connected network ($W_{e}$) is adopted to embed the dynamic elements into a high-dimensional vector space, as formulated in the following equation:

(27) $${de}_{n}=W_{e}\left(s_{n}\right),$$

where ${de}_{n}$ is the embedding form of $s_{n}$ at step *n*, and $W_{e}$ is the learnable parameter set of the fully connected network.

#### 3.3.4. Dynamic Encoder

The dynamic encoder is used for the further extraction of dynamic features. At every step, its inputs comprise the embedding form of the current state and the encoding form of the task that is selected at the previous step. The states of the AOS have the following characteristics: first, it is dynamically changing; second, the current state is affected by the previous one; third, the state sequence is time related. Hence, the dynamic encoder is composed of an LSTM cell ($L_{d}$), a concatenating operator, and a fully connected network ($W_{c}$). At step *n*, ${de}_{n}$ converts to a feature vector ${dc}_{n}$ through the LSTM cell at first. Then, the concatenation of ${dc}_{n}$ and ${sc}_{i_{n}}$ is transformed to $c_{n}$ by a fully connected network. This process is formulated as follows:

(28) $$dc_{n}=L_{d}\left(de_{n}\right),$$

(29) $$c_{n}=W_{c}\left(\mathrm{Concat}({sc}_{i_{n}},dc_{n}))\right),$$

where $L_{d}$ is the learnable parameter set of the LSTM cell, and $W_{c}$ is the learnable parameter set of the fully connected network.

#### 3.3.5. Decoder

The decoder makes decisions based on the outputs of the static encoder and the dynamic encoder, and it contains three components: an LSTM cell, a local attention layer, and a single-step scheduler.

At decoding step *n*, the LSTM cell ($L_{c}$) is adopted to process the outputs of the dynamic encoder at first owing to it appearing as the sequential characteristic, which is formulated as below:

(30) $$h_{n}=L_{c}\left(c_{n}\right),$$

where $L_{c}$ is the learnable parameter set of the LSTM cell.

Then, the designed local attention layer calculates the probability distribution of the candidate tasks according to the hidden state of the LSTM cell and the outputs of the static encoder, which are formulated in Equations (20) and (21), and the task with the highest probability will be selected to be scheduled. In this process, the range of the candidate tasks is narrowed, and the local attention mechanism only calculates the probability distribution of the tasks in this range. Significantly, the static encoding results of each task contain global feature information owing to the multi-head self-attention mechanism so that the task selection can tend toward global optimization.

Finally, the single-step scheduler selects the task with the highest probability and sets the earliest feasible time as its observation start time, and the single-step scheduling result is generated according to the complex constraints formulated in Section 2.3. Once the task is successfully executed, the rest of the unselected tasks will be judged on whether they have VTWs in the remaining period, and those without VTWs will be deleted, whose probability will be recorded.

### 3.4. Training Method

An actor–critic algorithm with an adaptive learning rate is adopted to train the proposed neural network, as presented in Algorithm 1. The actor–critic algorithm and the adaptive learning rate strategy are elaborated in detail below.
**Algorithm 1** Training algorithm 1: Initialize actor network parameters *θ* 2: Initialize critic network parameters *θ_c_* 3: Set actor network learning rate *lr*^1^ 4: Set critic network learning rate *lr_c_* 5: Training step counter *κ* ← 1 6: **for** *epoch* ← 1, ⋯, *Epoch* **do** 7:   **for** *task*_*num* ← 200, 150, 100, 50 **do** 8:    **for** *bn* ← 1, ⋯, *BN* **do** 9:     **for** *b* ← 1, ⋯, *BS* **do**10:      Decoding step counter *n* ← 011:      **while** not terminated **do**12:       Choose next task $ty_{n+1}^{b}\leftarrow \underset{ty}{\arg\max}\;P\left(ty|Y_{n}^{b},X_{n}^{b};\theta \right)$13:       Update  $X_{n+1}^{b}\leftarrow f\left(ty_{n+1}^{b},X_{n}^{b}\right)$14:       *n* ← *n* + 115:      **end while**16:      Calculate reward $R_{b}^{\kappa }$17:      Obtain evaluation value $V_{b}^{\kappa }$ through critic network18:     **end for**19:     $d\theta \leftarrow \frac{1}{BS}\sum_{b=1}^{BS}\left(R_{b}^{\kappa }-V_{b}^{\kappa }\right)\nabla_{\theta }\log P\left(Y^{b}|X_{0}^{b};\theta \right)$20:     $d\theta_{c}\leftarrow \frac{1}{BS}\sum_{b=1}^{BS}\nabla_{\theta_{c}}{\left(R_{b}^{\kappa }-V_{b}^{\kappa }\right)}^{2}$21:     Update *θ* ← *Adam*(*θ*, *dθ*)22:     Update *θ_c_* ← *Adam*(*θ_c_*, *dθ_c_*)23:     **if** *κ* mod 10 = 0 **then**24:      Update ${lr}^{\kappa +1}$ according to Equation (34)25:     **else**26:      $lr^{\kappa +1}\leftarrow \gamma \cdot lr^{\kappa }$27:     **end if**28:     *κ* ←*κ* + 129:    **end for**30:   **end for**31: **end for**

#### 3.4.1. Actor–Critic Algorithm

The actor–critic training algorithm is a common training algorithm for the optimization of neural networks [32], and it comprises two neural networks: an actor network and a critic network. The actor network is the proposed neural network for generating the scheduling result, and the profit ratio of the scheduling result is the reward of the actor network. The critic network is a separate network for evaluating the reward of inputs. Both of these networks need to be trained. In this study, the critic network is composed of an embedding layer, an encoder, and a decoder. Its embedding layer and encoder employ the same architecture as the actor network, while its decoder consists of two one-dimensional convolutional layers.

Algorithm 1 shows the pseudo-code of the training algorithm. The neural networks are trained on four kinds of samples with different task sizes *Epoch* times, which are introduced in Section 4.1. Each kind of sample contains *BN* batches. At the training step *κ*, given a batch of samples whose size is *BS*, the optimization process of the neural networks can be divided into five steps: (1) the actor network generates solutions through step-by-step construction; (2) the reward of every solution is calculated, and $R_{b}^{\kappa }$ is the reward of the solution *b*; (3) the critic network generates the evaluation value of every sample, and $V_{b}^{\kappa }$ is the evaluation value of the sample *b*; (4) the policy gradient of the actor network is calculated according to Equation (31), and that of the critic network is formulated in Equation (32); (5) Adam optimizer is adopted to optimize the parameters of the two networks according to the corresponding policy gradients and learning rates. The learning rate of the actor network is adjusted dynamically through the adaptive learning rate strategy, and that of the critic network is fixed.

(31) $$d\theta =\frac{1}{BS}\sum_{b=1}^{BS}\left(R_{b}^{\kappa }-V_{b}^{\kappa }\right)\nabla_{\theta }\log P\left(Y^{b}|X_{0}^{b};\theta \right).$$

(32) $$d\theta_{c}=\frac{1}{BS}\sum_{b=1}^{BS}\nabla_{\theta_{c}}{\left(R_{b}^{\kappa }-V_{b}^{\kappa }\right)}^{2}.$$

#### 3.4.2. Adaptive Learning Rate Strategy

In the training process of the neural network, the learning rate is a crucial hyperparameter that determines the update stride of network parameters. A large learning rate may lead to easy divergence, while a small learning rate may lead to slow convergence [41]. In the prior experiments, the conventional fixed learning rates and exponential learning rates failed to balance the early exploration and the later convergence, and a slightly higher learning rate could bring the reward curve to a higher level but make it difficult to converge. Therefore, the learning rate should be adjusted dynamically, and its appropriate decay and increase can make the reward curve converge to a higher level.

To improve the training effect of the actor network, an adaptive learning rate strategy is proposed that dynamically adjusts the learning rate according to the training performance. The reward curve can intuitively reflect the training performance. However, the neural network is trained on the four kinds of samples with significantly different optimal rewards, resulting in the reward curve appearing as a step shape. Hence, the learning rate cannot be adjusted according to the reward value, but the trend of the reward curve can be selected as the indicator for adjusting the learning rate. The adjustment process of the adaptive learning rate contains two steps: first, the learning rate overall decays by a fixed decay rate to ensure the convergence of the reward curve; then, for every ten training steps, if the trend of the reward curve does not reach the expected value, the learning rate will increase slightly to help the reward curve reach a higher level. After the learning rate is initialized or increases, the reward curve may be at a lower level in the early stage, but it is expected to rise rapidly in the middle stage of the following training and converge stably in the later stage, similar to a cosine wave of half a cycle. Thus, a cosine curve is used as the reference curve, and the expected value is calculated according to its slope. Details of the adaptive learning rate strategy are elaborated below.

Firstly, some variables need to be defined to describe the adaptive learning rate: at training step *κ*, given a batch of samples, (1) $lr^{\kappa }$ is the learning rate of the actor network; (2) $R^{\kappa }=\sum_{b=1}^{BS}R_{b}^{\kappa }$ is the average reward of this batch of solutions, denoting the reward at the current step; (3) given $\left\{R^{\kappa +\tau -11}|\tau =1,\cdots ,10\right\}$ which is the reward set of the previous ten steps, $\bar{R^{\kappa }}$ is its mean value; (4) $V^{\kappa }=\sum_{b=1}^{BS}V_{b}^{\kappa }$ is the average evaluation value of this batch of samples, denoting the evaluation value at the current step; (5) $\bar{V^{\kappa }}$ is the mean value of $\left\{V^{\kappa +\tau -11}|\tau =1,\cdots ,10\right\}$; (6) $sl^{\kappa }$ is the slope of the reward curve; (7) $e^{\kappa }$ represents the expected slope; (8) if $sl^{\kappa }<e^{\kappa }$, *κ* will be recorded as $\hat{\kappa }$ whose initial value is zero. (9) *K* is the total training steps; and (10) *γ* is the decay rate.

**Figure 6:**
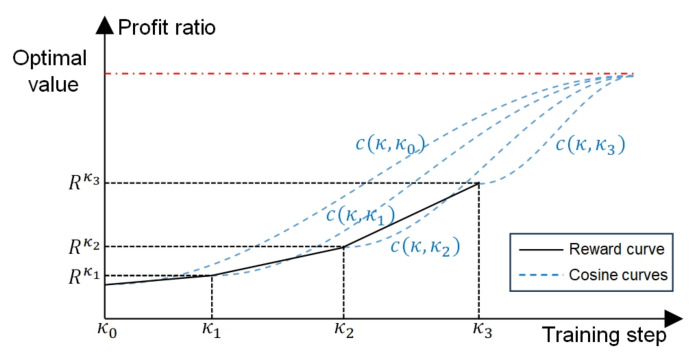
Diagram of the reward curve and the reference cosine curves. In this diagram, a fold line is used to represent the reward curve. However, the actual reward curve is a fluctuating curve. The appropriate increase in the learning rate can accelerate the rise of the reward curve in the mid-term of training. A new cosine curve is set as the reference cosine curve whenever the learning rate increases.

Secondly, the trend of the reward curve is expected to be close to that of a cosine curve, as shown in Figure 6. After ten training steps, the slope of the reward curve is compared with the expected value, which is gained according to the slope of the reference cosine curve. If the slope of the reward curve is less than the expected value, the learning rate of the actor network will increase slightly; otherwise, the learning rate will keep decaying at the following ten training steps. Once the learning rate increases, the reward curve is expected to rise like a new cosine curve at the following training steps.

Thirdly, the reference cosine curve function $c(\kappa ,\hat{\kappa })$ is formulated in Equation (33), and its derivative is formulated in Equation (34). The derivative function cannot be directly set as the expected slope for two reasons: (1) $V^{\hat{\kappa }}$ is just an evaluation value and not the actual optimal value, and it may be inaccurate and larger than 1; (2) $V^{\hat{\kappa }}$ and $R^{\hat{\kappa }}$ are the values at one step and cannot accurately represent training results. Thus, some improvements are made based on the derivative function. First, $|V^{\hat{\kappa }}-R^{\hat{\kappa }}|$ is replaced with $\frac{|V^{\hat{\kappa }}-R^{\hat{\kappa }}|}{V^{\hat{\kappa }}}$ to ensure the outcome is between 0 and 1. Then, $V^{\hat{\kappa }}$ and $R^{\hat{\kappa }}$ are, respectively, replaced with the mean values $\bar{V^{\kappa }}$ and $\bar{R^{\kappa }}$. In addition, $2\cdot (K-\hat{\kappa })$ is replaced with *K*, resulting in that the expected slope can be larger in the early stage of training and be smaller in the later stage. In this way, the learning rate can have more chances to rise in the early stage but can keep decaying in the later stage. The final expected slope $e^{\kappa }$ is formulated in Equation (35) through these improvements.

(33) $$c\left(\kappa ,\hat{\kappa }\right)=R^{\hat{\kappa }}+\frac{|V^{\hat{\kappa }}-R^{\hat{\kappa }}|}{2}\cdot \left[1-\cos\left(\frac{\kappa -\hat{\kappa }}{K-\hat{\kappa }}\pi \right)\right].$$

(34) $$\frac{dc(\kappa ,\hat{\kappa })}{d\kappa }=\frac{\pi \cdot |V^{\hat{\kappa }}-R^{\hat{\kappa }}|}{2\cdot (K-\hat{\kappa })}\cdot \sin\left(\frac{\kappa -\hat{\kappa }}{K-\hat{\kappa }}\pi \right).$$

(35) $$e^{\kappa }=\frac{\pi \cdot |\bar{V^{\kappa }}-\bar{R^{\kappa }}|}{K\cdot \bar{V^{\kappa }}}\cdot \sin\left(\frac{\kappa -\hat{\kappa }}{K-\hat{\kappa }}\pi \right).$$

Fourthly, the reward curve slope $sl^{\kappa }$ is calculated according to the reward values of the previous ten steps through the least square method, which is formulated as outlined below:

(36) $$sl^{\kappa }=\frac{\sum_{\tau =1}^{10}\left(\tau -\bar{\tau }\right)\left(R_{a}^{\kappa +\tau -11}-\bar{R^{\kappa }}\right)}{\sum_{\tau =1}^{10}{\left(\tau -\bar{\tau }\right)}^{2}},$$

where $\bar{\tau }$ is the mean value of one to ten.

Eventually, the adaptive learning rate $lr^{\kappa +1}$ of the next step is calculated as follows:

(37) $$lr^{\kappa +1}=\left\{\begin{array}{cc} \gamma \cdot lr^{\kappa }, & \kappa \;\mathrm{mod}\;10\neq 0\\ fl(lr^{\kappa }), & \kappa \;\mathrm{mod}\;10=0 \end{array}\right.,$$

where

(38) $$fl\left(lr^{\kappa }\right)=\left\{\begin{array}{cc} \gamma \cdot lr^{\kappa }, & sl^{\kappa }\geq e^{\kappa }\\ lr^{\kappa }+\frac{|\bar{V^{\kappa }}-\bar{R^{\kappa }}|}{\bar{V^{\kappa }}}\cdot \left(lr^{\hat{\kappa }}-lr^{\kappa }\right), & sl^{\kappa }<e^{\kappa } \end{array}\right..$$

As formulated in Equations (37) and (38), if the step number *κ* is the multiple of 10 and the reward curve slope $sl^{\kappa }$ is less than the expected value $e^{\kappa }$, the learning rate will increase slightly; otherwise, the learning rate $lr^{\kappa }$ will decay by the decay rate *γ*. As formulated in Equation (38), the increasing extent of the learning rate is determined by the difference between the evaluation value and the reward value and the difference between the last increased learning rate and the current one, and the learning rate cannot exceed the last increased learning rate. Through the adaptive learning rate strategy, the learning rate shows a downward trend but rises slightly at a few training steps, improving the exploration and convergence of the training algorithm.

### 3.5. Complexity Analysis

In the actor network, the time complexity of the static embedding layer is O(|T|), and that of the static encoder is $O(|T{|}^{2})$. At the decoding step *n*, the time complexity of the dynamic embedding layer and that of the dynamic encoder are both O(1), and that of the decoder is $O(|LS_{n}|)$. The time complexity of the *N*-step decoding process is formulated as follows:

(39) $$TC_{dp}=N\cdot TC_{del}+N\cdot TC_{de}+\sum_{n=1}^{N}|LS_{n}|\cdot TC_{d}=O(\sum_{n=1}^{N}|LS_{n}\left|\right),$$

where $TC_{del}$ is the computation time of the dynamical embedding layer at a step, $TC_{de}$ is the computation time of the dynamical encoder at a step, and $TC_{d}$ is the computation time of the decoder at a step. $TC_{del}$, $TC_{de}$, and $TC_{d}$ are constants. Since N≤|T| and $\left|LS_{n}\right|\leq \left|T\right|$, the time complexity of the actor network is $O(|T{|}^{2})$, which is formulated in Equation (40). The time complexity of generating a solution through the well-trained actor network is also $O(|T{|}^{2})$.

(40) $$TC_{actor}=O\left(|T|\right)+{O(|T|}^{2})+O(\sum_{n=1}^{N}|LS_{n}\left|\right)={O(|T|}^{2}).$$

In the critic network, the time complexity of its embedding layer is O(|T|), that of its encoder is $O(|T{|}^{2})$, and that of its decoder is O(|T|). Thus, the time complexity of the critic network is $O(|T{|}^{2})$, which is formulated in Equation (41).

(41) $$TC_{critic}=O\left(|T|\right)+{O(|T|}^{2})+O(|T|)={O(|T|}^{2}).$$

In the training process, the time complexity of training once for a batch of samples is formulated in Equation (42). And solving an SAOSSP only needs the actor network to run once, so the time complexity of solving is also $O(|T{|}^{2})$.

(42) $$TC_{training}=BS\cdot \left(TC_{actor}+TC_{critic}\right)={O(BS\cdot |T|}^{2}).$$

From the above analysis, the training time and solving time of DRLLA rise polynomially as the task size |T| increases. In addition, the training time is also directly affected by the training times, the number of sample types, and the batch size.

## 4. Computational Experiments

In this section, a dataset of the SAOSSP with different task scales is created to train and test the proposed neural network. Then, sufficient experiments are carried out to verify the effectiveness of the proposed algorithm. The main aspects of validation include the training performance, the adaptability to various-scale instances, and the effect of the local attention mechanism and the adaptive learning rate strategy. The experiments are conducted on a laptop computer with Intel(R) Core (TM) i7-7700HQ CPU @ 2.80 GHz and 40 GB RAM. The DRL framework embedded in Pytorch 1.5.1 in Python 3.8 is adopted in this study.

### 4.1. Dataset

Due to the lack of public datasets, a large number of instances are designed referring to Reference [4]. In these instances, the orbital parameters of the AOS are listed in Table 2, and its other attribute parameters are listed in Table 3. Numerous tasks are randomly distributed in different areas. For each task, the requested observing duration is a random integer between 5 and 10, and the minimum image quality and priority are both random integers between 1 and 10, which are listed in Table 4. The scheduling time horizon is from 1 January 2023, 00:00:00, to 1 January 2023, 24:00:00. With the growth in the task number, the tasks are distributed more densely in the region, aggravating conflicts among tasks, and only partial tasks have a chance to be completed.

In order to enhance and verify the adaptability of the proposed algorithm, it is necessary to ensure the diversity of the training and testing datasets, whose details are shown in Table 5. The training dataset Training_R1 contains four kinds of training samples, each with a quantity of 2560. Tasks in the training samples are distributed in a region with the latitude from 3° N to 53° N and the longitude from 73° E to 133° E. Four testing datasets with tasks located in different areas are created to verify the effectiveness of the proposed algorithm, including Testing_R1, Testing_R2, Testing_R3, and Testing_G. Each of them contains five types of testing samples, each with a quantity of 128. The datasets can be publicly available at https://github.com/neverlinever/Dataset_AOSSP.git (20 August 2024).

### 4.2. Training Process

The parameter settings of the DRLLA are listed in Table 6, containing the network parameters and the training parameters. The total number of training epochs is 10. In every epoch, the network is trained on the training dataset Training_R1 in the order of 200-task, 150-task, 100-task, and 50-task samples, resulting in its reward curve in the training process appearing in a stepped shape. One training epoch contains 80 training steps because of the training parameter settings.

**Figure 7:**
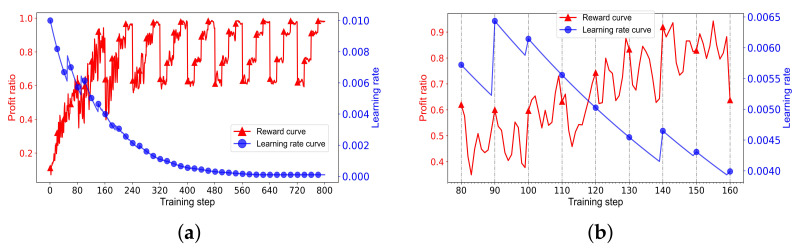
Training process of DRLLA. (**a**) The reward curve and the learning rate curve in the whole training process. (**b**) The reward curve and the learning rate curve in the second training epoch.

The training process is shown in Figure 7. As depicted in Figure 7a, the reward curve fluctuates sharply in the first three training epochs but trends toward stability and convergence in the last three training epochs. The learning rate curve overall shows a downward trend but rises multiple times.

Figure 7b shows the details of the training process in the second training epoch (from the 80th training step to the 160th training step), and the learning rate rises obviously five times in this epoch. It can be seen from these two figures that the adaptive learning rate strategy can balance the early exploration and the later convergence of the actor network and the actor network is trained well through the actor–critic training algorithm with the adaptive learning rate.

### 4.3. Comparison with the State-of-the-Art Algorithms

To verify the effect of DRLLA, we compare it with seven state-of-the-art algorithms for satellite scheduling problems or other combination optimization problems, including Transformer (TRFM) [42], PtrNet [31], RLPT [32], a reinforcement learning-based genetic algorithm (RLGA) [43], a hybrid discrete artificial bee colony algorithm (HDABC) [21], an improved simulated annealing algorithm (ISA) [37], and an improved ant colony optimization algorithm (IACO) [44]. TRFM, PtrNet, and RLPT are neural network models with encoder–decoder structures, and RLGA, HDABC, ISA, and IACO are heuristic algorithms with improved search mechanisms. The heuristic algorithms maintain their original settings. The comparison experiments contain two parts: first, these algorithms are tested on the dataset Testing_R1 to validate the effectiveness of DRLLA under different problem scales; then, they are tested on the other three datasets Testing_R2, Testing_R3, and Testing_G, whose task distribution is different from Testing_R1, to demonstrate the generalization ability of DRLLA in various instances.

**Figure 8:**
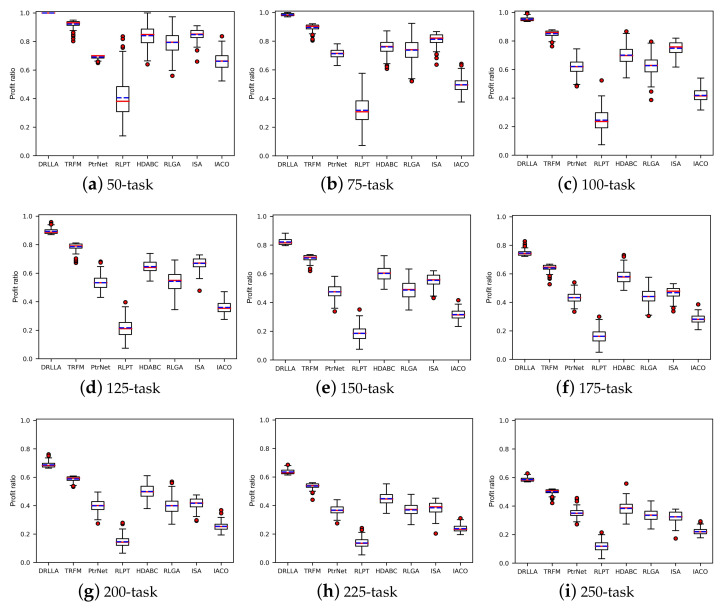
Profit ratio distributions of different algorithms on the dataset Testing_R1.

**Figure 9:**
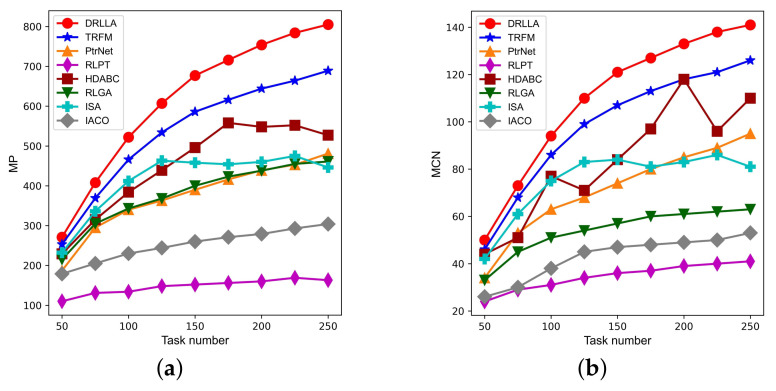
Curves of mean profit and mean completion number. (**a**) Mean profit. (**b**) Mean completion number.

Figure 8 and Figure 9 and Table 7 show the detailed testing results of the first comparison experiment on the testing dataset Testing_R1. The boxplot graphs in Figure 8 illustrate the profit ratio distribution of solutions obtained by different algorithms. In these boxplot graphs, the top lines denote maximum values, the bottom lines denote minimum values, the middle boxes denote interquartile ranges, the red lines denote medians, the blue dashed lines denote mean values, and the red circles denote outliers. As shown in these boxplot graphs, the profit ratio distributions of DRLLA are highly concentrated, and its median lines are higher than the comparison algorithms, demonstrating that DRLLA can obtain solutions with the highest profit ratio when handling various AOS scheduling instances. The profit ratio distributions of TRFM are also concentrated, and its median lines are higher than other comparison algorithms but lower than DRLLA. The profit ratio distributions of the remaining algorithms are relatively scattered. In general, the stability and generalization of DRLLA and TRFM are superior to the remaining comparison algorithms, and DRLLA can obtain solutions with higher profit ratios than the comparison algorithms.

Table 7 presents the detailed testing results of the first comparison experiment on the dataset Testing_R1. The indicators include the mean profit ratio (MPR), mean profit (MP), mean completion number (MCN), and mean computational time (MCT), and the best values are bolded. Figure 9 depicts the MP curves and MCN curves of different algorithms. The testing results are presented as follows:

(1) As for the DRL-based algorithms, DRLLA can obtain better solutions with the higher profit and completion number than these comparison algorithms in a relatively small amount of computational time under different task scales; TRFM, only second to DRLLA, can also gain acceptable solutions with a little more computational time; although RLPT has the shortest computational time in these testing cases, it is hard to achieve satisfactory solutions.
(2) Among the heuristic algorithms, ISA and HDABC can obtain relatively acceptable solutions, but the computational time of HDABC far exceeds other algorithms; RLGA and IACO perform worse than the other algorithms in terms of solution quality. Comparing with the DRL-based algorithms, these heuristic algorithms need more computational time due to the huge computation burden of population iteration.
(3) As the task number increases, the MPR values of all algorithms continuously decrease, while the MP and MCN values of most algorithms keep growing. The main reason is that the increases in the total profit and the total task number are more significant than in the gained profit and the completion number, indicating that the problem scales exceed the observation capability of the AOS.
(4) In general, DRLLA shows excellent performance in solution quality over the comparison algorithms. With the increase of task scale, the gap of the MPR values between DRLLA and the comparison algorithms becomes significant. In addition, the computational time of DRLLA is not less than that of RLPT, but its computation efficiency is superior to other comparison algorithms, especially heuristic algorithms. Thus, the comparison results on the dataset Testing_R1 fully demonstrate that DRLLA is capable of obtaining high-quality solutions within acceptable computational time.

To further validate the generalization ability of the proposed algorithm on various instances, we conduct comparison experiments of DRLLA and the comparison algorithms on three other testing datasets Testing_R2, Testing_R3, and Testing_G, and the testing results are presented in Table 8, where the best values are bolded. HDABC is not used as the comparison algorithm because of its high time consumption. According to the comparison results, DRLLA exhibits significant advantages over the comparison algorithms in terms of solution quality on these three testing datasets. In terms of computational time, the DRL-based algorithms outperform the heuristic algorithms due to the different solution constructions, and the computational time of DRLLA is far less than that of the heuristic algorithms. The superior testing performance of DRLLA on the different datasets fully demonstrates that it has excellent generalization ability to be extended for various SAOSSP instances.

### 4.4. Ablation Experiments

DRLLA consists of two main parts: the neural network and the training algorithm. In the proposed neural network, a multi-head self-attention mechanism is introduced to the static encoder for extracting task features, and the local attention mechanism is applied to decoding for constructing the scheduling solutions. In the training algorithm, the adaptive learning rate strategy is adopted to improve the training effect. In this subsection, ablation experiments are performed to further validate their effectiveness.

#### 4.4.1. Effectiveness Verification of the Multi-Head Self-Attention Mechanism

To demonstrate the contribution of the multi-head self-attention (MHSA), we, respectively, adopt a convolutional neural network (CNN) and LSTM to extract features in the static encoder to build comparison neural network models. The kernel size of CNN is set as 5. The comparison network models are trained first on the dataset Training_R1 and then tested on the dataset Testing_R1.

Table 9 presents the testing results of the proposed network with MHSA and the comparison networks with CNN and LSTM, and the best values are bolded. It can be seen that MHSA performs better than CNN and LSTM in terms of MPR, and it takes the least computational time in the instances with 150, 200, and 250 tasks, indicating that the features extracted by MHSA are more beneficial for the subsequent solution construction. The main reason is that their feature extraction techniques are different. CNN uses a convolution kernel to scan the task information to extract local features, and the extracted features of a task are related to its own and adjacent tasks. LSTM processes task information in a sequencing order, and the extracted features of a task are relevant to its own and the previous tasks. As for CNN and LSTM, the sequencing order of the input tasks can influence the feature extraction results. In comparison, MHSA adopts multiple independent self-attention heads to extract diverse features, and each head focuses on different parts of the task sequence that are not related to the task order. Thus, MHSA has significant advantages over CNN and LSTM in feature extraction.

#### 4.4.2. Effectiveness Verification of the Local Attention Mechanism

To demonstrate the contribution of the local attention mechanism, we replace it with the global attention mechanism to build a comparison network, train the comparison network with the same training algorithm, and compare the testing results of the two algorithms on the dataset Testing_R1. This deep reinforcement learning algorithm with the global attention mechanism is denoted by DRLGA.

Table 10 lists the testing results of DRLLA and DRLGA, where the best values are bold. As for MPR, DRLLA is significantly better than DRLGA, indicating that the local attention mechanism is superior to the global attention mechanism in improving solution quality. The main reason is that the local attention mechanism, in comparison with the global attention mechanism, can guide the network model to focus on the more promising candidate task range, preventing it from generating unreasonable task permutations. However, the MCT values of DRLGA are much lower than those of DRLLA. The main reason is that the global attention mechanism tends to generate inappropriate task permutations, leading some unscheduled tasks to lose their observation opportunities and reducing the number of decoding steps. This mechanism shortens the computation time but seriously sacrifices the solution quality. In comparison, the local attention mechanism takes more computational time, but it dramatically improves the solution quality.

#### 4.4.3. Effectiveness Verification of the Adaptive Learning Rate Strategy

To validate the feasibility and effectiveness of the adaptive learning rate strategy, we compare the proposed DRLLA with the adaptive learning rate (DRLLA-a) to two compared algorithms: DRLLA with a fixed learning rate of 0.001 (DRLLA-f), and DRLLA with an exponential learning rate (DRLLA-e). In DRLLA-e, the initial learning rate is 0.01, and the decay rate is 0.001. The three algorithms are trained on the dataset Training_R1 first and then tested on the dataset Testing_R1. The comparison results of training and testing are presented as follows.

**Figure 10:**
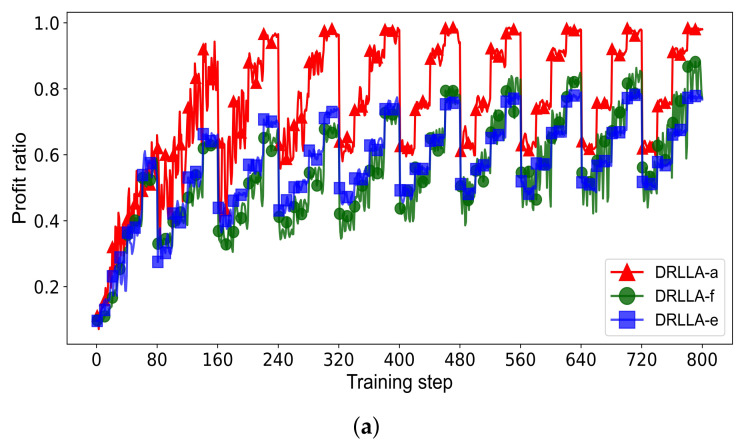

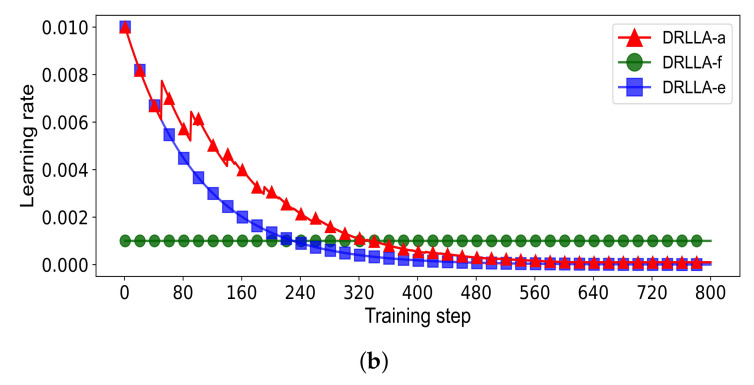
Training process of DRLLA-a, DRLLA-f, and DRLLA-e. (**a**) Profit ratio curves of DRLLA-a, DRLLA-f, and DRLLA-e in the training process. (**b**) Learning rate curves of DRLLA-a, DRLLA-f, and DRLLA-e in the training process.

Figure 10 depicts the training process of these three algorithms, including their profit ratio curves and learning rate curves. It can be observed that DRLLA-a performs best among these algorithms in the training process. Its profit ratio curve is similar to those of the comparison algorithms in the first training epoch but quickly reaches a higher level in the following training epochs owing to the appropriate rise of the learning rate. In the later stage of training, its learning rate decreases to a low level, and its profit ratio curve tends to be stable and convergent. DRLLA-e can achieve a little better profit ratio than DRLLA-f in the early stage of training, while the opposite is true in the later stage of training. The profit ratio curve of DRLLA-e is more stable than that of DRLLA-f, especially in the later stage. The training results indicate that (1) a high learning rate can improve the exploration ability of the network model in the early stage of training, while a low learning rate can improve its stability and convergence in the later stage of training; (2) according to such characteristics, the proposed adaptive learning rate strategy appropriately increases the learning rate on the basis of the exponential learning rate, enhancing the early exploration ability and the later convergence ability of the training algorithm.

Table 11 lists the testing results of the three algorithms, where the best values are bolded. The standard deviation of profit ratios (STD) is set as an additional comparison indicator, and the lower STD value indicates the more densely distributed solutions and further demonstrates the better stability and generalization of the algorithm. DRLLA-a can reach the highest MPR value within the acceptable computational time in most testing instances, and its STD value, close to that of DRLLA-e, is significantly better than that of DRLLA-f. As for DRLLA-f, its MPR value is slightly lower than that of DRLLA-a in most testing instances and even exceeds that of DRLLA-a when the task number is 250, while it performs the worst in terms of STD. DRLLA-e has superior stability ability and takes the least computational time, but it fails to generate solutions with high profit ratios. The testing results demonstrate that the proposed neural network trained with the adaptive learning rate training algorithm has an excellent solving ability, and the comprehensive testing performance of DRLLA-a is superior to the compared algorithms.

## 5. Conclusions and Future Work

In this paper, a deep reinforcement learning algorithm with a local attention mechanism is proposed to address the scheduling problem of a single agile optical satellite with different task scales. Two techniques are effectively adopted to improve the performance of the algorithm: (1) local attention mechanism and (2) adaptive learning rate strategy. The local attention mechanism narrows the range of candidate tasks and selects the next-scheduled task from a more promising range, significantly improving the quality of the generated solution. The adaptive learning rate strategy dynamically decreases or increases the learning rate according to the reward curve trend in the training process, enhancing the early exploration and later convergence abilities. Based on these techniques, the proposed algorithm exhibits superior performance in solution quality, generalization, and efficiency in comparison with the state-of-the-art algorithms. The experimental results also validate the effectiveness of the local attention mechanism for generating high-quality solutions and the adaptive learning rate strategy for improving the training effect.

This paper can provide an efficient and effective approach for agile optical satellite scheduling, while there are still some areas for improvement. Some practical factors are not fully considered, such as the influence of cloud cover, lighting conditions, and observation angles on the imaging results. For future work, we will give a comprehensive consideration of various practical constraints and establish a more realistic AOS scheduling model. In addition, we will further improve the proposed algorithm to make it more available for practical application, and the algorithm will be extended to more complex instances, such as multi-AOS scheduling, emergency scheduling of AOSs, and uncertainty scheduling of AOSs.

## Figures and Tables

**Table 1:** Variable definition.

Variable	Definition
*op*	Orbital parameters of the AOS
$O=\{o_{l}|1\leq l\leq |O|\}$	Set of orbits, a total of |*O*| orbits
$OL(o_{l})$	Period which meets the light condition in the orbit $o_{l}$
*M*	Maximum memory used for image storage
*E*	Maximum energy used for observation and attitude adjustment
rm	Memory consumption rate when the AOS observes
reo	Energy consumption rate when the AOS observes
rea	Energy consumption rate when the AOS adjusts its attitude
$\left[r_{min},r_{max}\right]$	Range of the observation roll angle, $r_{min}=-r_{max}$
$\left[p_{min},p_{max}\right]$	Range of the observation pitch angle, $p_{min}=-p_{max}$
$T=\{t_{i}|i=1,\cdots ,|T|\}$	Set of tasks, a total of |T| tasks
${loc}_{i}=\left({lat}_{i},{lon}_{i}\right)$	Location of the task $t_{i}$, ${lat}_{i}$ is its latitude and ${lon}_{i}$ is its longitude
$q_{i}$	Minimum image quality requirement of the task $t_{i}$, whose value is divided into ten levels from 1 to 10
$d_{i}$	Required observation duration of the task $t_{i}$
$p_{i}$	Priority of the task $t_{i}$, representing the observation profit
$VTW=\{W_{i}|i=1,\cdots ,|T|\}$	VTW set of all tasks
$W_{i}=\{w_{i}^{k}\left|k=1,\cdots ,\right|W_{i}\left|\right\}$	VTW set of the task $t_{i}$, a total of $\left|W_{i}\right|$ VTWs
$w_{i}^{k}=\left[c_{i}^{k},{st}_{i}^{k},{et}_{i}^{k},r_{i}^{k}\right]$	The *k*th VTW of the task $t_{i}$
$c_{i}^{k}$	Orbit index of the VTW $w_{i}^{k}$
${st}_{i}^{k}$	Start time of the VTW $w_{i}^{k}$
${et}_{i}^{k}$	End time of the VTW $w_{i}^{k}$
$ra_{i}^{k}$	Roll angle in the VTW $w_{i}^{k}$
*A*	Observation action sequence of the AOS
$a_{i}^{k_{i}}\in A$	Observation action when the AOS executes the task $t_{i}$ in the VTW $w_{i}^{k_{i}}$
${ost}_{i}^{k_{i}}$	Observation start time of the observation action $a_{i}^{k_{i}}$
${oet}_{i}^{k_{i}}$	Observation end time of the observation action $a_{i}^{k_{i}}$, ${oet}_{i}^{k_{i}}=a_{i}^{k_{i}}+d_{i}$
${pa}_{i}^{k_{i}}$	Observation pitch angle of the observation action $a_{i}^{k_{i}}$
${mr}_{i}$	Rest memory after the task $t_{i}$ is executed
${er}_{i}$	Rest energy after the task $t_{i}$ is executed

**Table 2:** Orbital parameters of the AOS.

Semimajor Axis	Eccentricity	Inclination	Argument of Perigee	RAAN	True Anomaly
7200 km	6.24 × 10^−4^	97.576°	0°	140.72°	35.075°

**Table 3:** Attribute parameters of the AOS.

*M*	*E*	*rm*	*reo*	*rea*	*r_max_*	*r_min_*	*p_max_*	*r_min_*	*ω*	*a*	*ST*
1000	1500	1	1	0.5	45	−45	45	−45	3	1	1

**Table 4:** Attribute parameters of tasks.

Notion	Definition	Type	Value Range
*q*	Minimum image quality	Integer	[1, 10]
*d*	The requested observation duration	Integer	[5, 10]
*p*	Priority	Integer	[1, 10]

**Table 5:** Details of the training and testing datasets.

Dataset	Task Distribution Type	Latitude Range	Longitude Range	Number of Tasks	Number of Instances for Each Type
Training_R1	Regional distribution	[3° N, 53° N]	[73° E, 133° E]	50, 100, 150, 200	2560
Testing_R1	Regional distribution	[3° N, 53° N]	[73° E, 133° E]	50, 75, 100, 125, 150, 175, 200, 225, 250	128
Testing_R2	Regional distribution	[3° N, 53° N]	[3° E, 63° E]	50, 100, 150, 200, 250	128
Testing_R3	Regional distribution	[3° N, 53° N]	[63° W, 123° W]	50, 100, 150, 200, 250	128
Testing_G	Global distribution	[70° S, 70° N]	[180° W, 180° E]	50, 100, 150, 200, 250	128

**Table 6:** Parameter settings of DRLLA.

**Actor network**
Static embedding layer: Linear(input_size = 2, out_size = 128),
LSTMcell(input_size = 6, hidden_size = 128),
Linear(input_size = 256, out_size = 128);
Static encoder: MultiHeadAttention(embeded_dim = 128, num_heads = 2, dropout = 0.1),
Conv1d(input_size = 128, output_size = 128, kernel_size = 1);
Dynamic embedding layer: Linear(input_size = 6, out_size = 128);
Dynamic encoder: LSTMcell(input_size = 128, hidden_size = 128),
Linear(input_size = 256, out_size = 128);
Decoder: LSTMcell(input_size = 6, hidden_size = 128),
Local attention (which only considers the unselected tasks with VTWs in the current orbit or the next one at every decoding step).
**Critic network**
Embedding layer: the same as the static embedding layer;
Encoder: the same as the static encoder;
Decoder: Conv1d(input_size = 128, output_size = 64, kernel_size = 1),
Conv1d(input_size = 64, output_size = 16, kernel_size = 1),
Conv1d(input_size = 16, output_size = 128, kernel_size = 1).
**Training parameters**
Actor network: adaptive learning rate (whose initial value is *lr*^1^ = 0.1
and decay rate is *γ* = 0.999);
Critic network: fixed learning rate (*lr_c_* = 0.001);
Number of training epochs: *Epoch* = 10.

**Table 7:** Detailed testing results of different algorithms on the dataset Testing_R1.

Problem Scales	Indicators	DRLLA	TRFM	PtrNet	RLPT	HDABC	RLGA	ISA	IACO
50-task	MPR	**1.0**	0.922	0.713	0.406	0.841	0.793	0.848	0.661
	MP	**271**	250	187	110	229	215	232	179
	MCN	**50**	46	34	24	44	33	42	26
	MCT (s)	0.147	0.156	0.175	**0.049**	1706.386	32.484	11.932	4.492
75-task	MPR	**0.984**	0.896	0.691	0.317	0.760	0.737	0.813	0.496
	MP	**408**	369	295	131	315	305	336	205
	MCN	**73**	68	53	29	51	45	61	30
	MCT (s)	0.148	0.244	0.246	**0.079**	1842.016	44.052	20.463	8.900
100-task	MPR	**0.953**	0.851	0.621	0.245	0.701	0.627	0.749	0.419
	MP	**522**	466	340	134	384	343	412	230
	MCN	**94**	86	63	31	77	51	75	38
	MCT (s)	0.273	0.317	0.346	**0.103**	2033.880	55.394	22.037	15.256
125-task	MPR	**0.893**	0.786	0.534	0.217	0.646	0.543	0.669	0.360
	MP	**607**	534	363	148	439	368	463	244
	MCN	**110**	99	68	34	71	54	83	45
	MCT (s)	0.309	0.387	0.463	**0.144**	2123.224	67.529	34.638	20.787
150-task	MPR	**0.822**	0.710	0.474	0.185	0.603	0.486	0.554	0.316
	MP	**677**	585	390	152	496	400	458	260
	MCN	**121**	107	74	36	98	57	84	47
	MCT (s)	0.346	0.485	0.576	**0.182**	2571.790	77.264	45.204	32.148
175-task	MPR	**0.746**	0.641	0.433	0.162	0.581	0.441	0.468	0.282
	MP	**716**	616	416	156	558	423	454	271
	MCN	**127**	113	80	37	97	60	81	48
	MCT (s)	0.481	0.558	0.641	**0.222**	2721.985	86.827	55.720	40.752
200-task	MPR	**0.687**	0.588	0.400	0.146	0.500	0.399	0.417	0.255
	MP	**754**	644	439	160	548	438	460	279
	MCN	**133**	118	85	39	118	61	83	49
	MCT (s)	0.512	0.648	0.825	**0.311**	2818.767	95.474	67.816	51.379
225-task	MPR	**0.636**	0.537	0.367	0.137	0.448	0.369	0.384	0.237
	MP	**784**	663	453	169	552	455	475	293
	MCN	**138**	121	89	40	96	62	86	50
	MCT (s)	0.574	0.718	1.014	**0.382**	2897.828	104.961	76.227	73.606
250-task	MPR	**0.586**	0.501	0.350	0.119	0.384	0.336	0.324	0.221
	MP	**805**	689	481	163	527	461	446	304
	MCN	**141**	126	95	41	110	63	81	53
	MCT (s)	0.615	0.888	0.997	**0.452**	2995.907	114.868	87.382	79.007

**Table 8:** Testing results on the datasets Testing_R2, Testing_R3, and Testing_G.

Datasets	Scales	Indicators	DRLLA	TRFM	PtrNet	RLPT	RLGA	ISA	IACO
Testing_R2	50-task	MPR	**1.0**	0.983	0.959	0.491	0.792	0.835	0.532
		MCT (s)	0.195	0.210	0.357	**0.071**	32.954	12.461	4.468
	100-task	MPR	**0.946**	0.819	0.735	0.290	0.581	0.702	0.378
		MCT (s)	0.429	0.446	0.376	**0.149**	57.148	34.163	13.647
	150-task	MPR	**0.783**	0.668	0.558	0.224	0.475	0.547	0.310
		MCT (s)	0.513	0.496	0.692	**0.233**	79.459	53.917	28.781
	200-task	MPR	**0.605**	0.547	0.370	0.173	0.409	0.398	0.272
		MCT (s)	0.605	0.714	0.859	**0.337**	99.515	71.086	49.661
	250-task	MPR	**0.513**	0.476	0.274	0.143	0.367	0.307	0.245
		MCT (s)	0.773	0.963	1.104	**0.466**	103.148	89.937	76.245
Testing_R3	50-task	MPR	**1.0**	0.950	0.942	0.497	0.790	0.825	0.536
		MCT (s)	0.192	0.219	0.324	**0.071**	33.821	12.998	4.207
	100-task	MPR	**0.927**	0.790	0.757	0.298	0.575	0.697	0.376
		MCT (s)	0.412	0.464	0.509	**0.148**	58.458	35.867	13.647
	150-task	MPR	**0.759**	0.646	0.574	0.219	0.480	0.527	0.311
		MCT (s)	0.482	0.521	0.692	**0.234**	73.412	54.157	26.482
	200-task	MPR	**0.652**	0.533	0.343	0.172	0.408	0.417	0.271
		MCT (s)	0.586	0.740	0.810	**0.343**	93.024	69.022	48.329
	250-task	MPR	**0.560**	0.412	0.291	0.170	0.362	0.373	0.246
		MCT (s)	0.750	0.958	1.065	**0.464**	105.293	81.362	79.694
Testing_G	50-task	MPR	**0.941**	0.619	0.467	0.310	0.627	0.787	0.495
		MCT (s)	0.182	0.174	0.229	**0.074**	31.890	11.736	4.207
	100-task	MPR	**0.738**	0.526	0.308	0.261	0.505	0.648	0.328
		MCT (s)	0.239	0.451	0.441	**0.179**	47.403	30.683	14.383
	150-task	MPR	**0.668**	0.460	0.263	0.195	0.406	0.587	0.265
		MCT (s)	0.388	0.694	0.935	**0.290**	66.627	47.226	30.895
	200-task	MPR	**0.615**	0.420	0.243	0.151	0.352	0.445	0.232
		MCT (s)	0.491	0.825	1.116	**0.395**	87.145	58.114	49.884
	250-task	MPR	**0.572**	0.390	0.232	0.125	0.311	0.312	0.211
		MCT (s)	0.722	1.002	1.236	**0.574**	112.460	71.429	70.982

**Table 9:** Testing results of MHSA, CNN and LSTM on Testing_R1.

Task Number		50	75	100	125	150	175	200	225	250
MPR	MHSA	**1.0**	**0.984**	**0.953**	**0.893**	**0.822**	**0.746**	**0.687**	**0.636**	**0.586**
	CNN	0.983	0.947	0.895	0.826	0.760	0.683	0.632	0.578	0.537
	LSTM	0.981	0.957	0.926	0.878	0.818	0.727	0.656	0.608	0.567
MCT (s)	MHSA	0.147	0.148	0.273	0.309	0.346	0.481	0.512	0.574	0.615
	CNN	0.087	0.148	0.221	0.282	0.353	0.417	0.585	0.560	0.626
	LSTM	0.090	0.156	0.224	0.303	0.375	0.473	0.538	0.620	0.796

**Table 10:** Testing results of DRLLA and DRLGA on Testing_R1.

Task Number		50	75	100	125	150	175	200	225	250
MPR	DRLLA	**1.0**	**0.984**	**0.953**	**0.893**	**0.822**	**0.746**	**0.687**	**0.636**	**0.586**
	DRLGA	0.456	0.352	0.309	0.269	0.231	0.210	0.190	0.177	0.165
MCT (s)	DRLLA	0.147	0.148	0.273	0.309	0.346	0.481	0.512	0.574	0.615
	DRLGA	**0.045**	**0.039**	**0.052**	**0.079**	**0.114**	**0.110**	**0.131**	**0.184**	**0.236**

**Table 11:** Testing results of the neural networks trained by three training algorithms on Testing_R1.

Task Number		50	75	100	125	150	175	200	225	250
MPR	DRLLA-a	**1.0**	**0.984**	**0.953**	**0.893**	**0.822**	**0.746**	**0.687**	**0.636**	0.586
	DRLLA-f	0.924	0.890	0.852	0.810	0.766	0.720	0.673	0.632	**0.593**
	DRLLA-e	0.472	0.371	0.323	0.297	0.270	0.256	0.236	0.225	0.217
STD	DRLLA-a	**0**	**0.009**	**0.014**	0.019	0.021	0.019	0.020	**0.017**	**0.014**
	DRLLA-f	0.045	0.048	0.045	0.042	0.035	0.035	0.033	0.028	0.030
	DRLLA-e	0.017	0.018	0.016	**0.018**	**0.014**	**0.011**	**0.014**	0.018	0.015
MCT(s)	DRLLA-a	0.147	0.148	0.273	0.309	0.346	0.481	0.512	0.574	0.615
	DRLLA-f	0.083	0.140	0.212	0.278	0.356	0.445	0.545	0.652	0.685
	DRLLA-e	**0.048**	**0.066**	**0.086**	**0.114**	**0.137**	**0.167**	**0.193**	**0.220**	**0.257**

## Data Availability

Data are contained within the article.
